# Structural Arrangement Produced by Concanavalin A Binding to Homomeric GluK2 Receptors

**DOI:** 10.3390/membranes11080613

**Published:** 2021-08-11

**Authors:** Cuauhtemoc U. Gonzalez, Elisa Carrillo, Vladimir Berka, Vasanthi Jayaraman

**Affiliations:** 1 Center for Membrane Biology, Department of Biochemistry and Molecular Biology, University of Texas Health Science Center at Houston, 6431 Fannin St., Houston, TX 77030, USA; Cuauhtemoc.Gonzalez@uth.tmc.edu (C.U.G.); Elisa.CarrilloFlores@uth.tmc.edu (E.C.); Vladimir.Berka@uth.tmc.edu (V.B.); 2 MD Anderson Cancer Center UTHealth Graduate School of Biomedical Sciences, University of Texas Health Science Center at Houston, 6431 Fannin St., Houston, TX 77030, USA

**Keywords:** kainate receptor, Concanavalin A, smFRET, electrophysiology

## Abstract

Kainate receptors are members of the ionotropic glutamate receptor family. They form cation-specific transmembrane channels upon binding glutamate that desensitize in the continued presence of agonists. Concanavalin A (Con-A), a lectin, stabilizes the active open-channel state of the kainate receptor and reduces the extent of desensitization. In this study, we used single-molecule fluorescence resonance energy transfer (smFRET) to investigate the conformational changes underlying kainate receptor modulation by Con-A. These studies showed that Con-A binding to GluK2 homomeric kainate receptors resulted in closer proximity of the subunits at the dimer–dimer interface at the amino-terminal domain as well as between the subunits at the dimer interface at the agonist-binding domain. Additionally, the modulation of receptor functions by monovalent ions, which bind to the dimer interface at the agonist-binding domain, was not observed in the presence of Con-A. Based on these results, we conclude that Con-A modulation of kainate receptor function is mediated by a shift in the conformation of the kainate receptor toward a tightly packed extracellular domain.

## 1. Introduction

Kainate receptors belong to the family of cation-permeable channels known as ionotropic glutamate receptors (iGluRs). These receptors are activated by glutamate and are important in excitatory neuronal transmission throughout the central nervous system [1,2,3,4]. Kainate receptors, like other iGluRs, are tetrameric proteins arranged as a dimer of dimers in their extracellular segments. Furthermore, each subunit of the receptor can be separated into four major domains: the two extracellular domains, which comprise the amino-terminal domain (ATD) and the agonist-binding domain (ABD); the transmembrane segments; and the cytosolic carboxy-terminal domain [3,5,6]. It is through induced conformational changes occurring at these domains that agonists and other small molecules regulate channel activity [7,8,9].

Structural and spectroscopic measurements have provided significant insight into the conformational changes underlying kainate receptor activation and desensitization [6,7,9,10,11,12,13,14]. In the resting, apo state of the kainate receptor, when there is no glutamate bound, the cleft within the bi-lobed, agonist-binding domain is open, and there are extensive interactions (coupling) between the subunits forming the dimer of the ABD. The binding of glutamate leads to closure of the ABD cleft [13,15]. In the event of coupling across the ABD dimers, the cleft closure conformational change pulls on the transmembrane segments, moving them apart, resulting in opening of the channel pore and thus, allowing the influx of cations. Decoupling of the ABD dimer interface, on the other hand, allows for the release of stress at the transmembrane segments. This release in stress allows for the transmembrane segments to close, preventing further influx of cations and leading to receptor desensitization [6,7,9].

Exogenous jack-bean extract, Concanavalin A (Con-A), is a lectin which is known to bind to carbohydrates attached to proteins through *N*-glycosylation [16,17,18]. Con-A has been found to modulate the function of kainate receptors by changing the extent of desensitization [16,19]. Using site-directed mutagenesis, it has been shown that the glycosylation sites on kainate receptors play a role in Con-A modulation [17,18]. These glycosylation sites are spread throughout the extracellular ATD and ABD domains, and it has not been possible to draw direct correlations between binding site, conformation, and functional modulation [17,18,20,21]. Functional studies have shown that Con-A binding and modulation is state-dependent. Specifically, preincubation with Con-A prior to glutamate application produces larger effects on loss of desensitization than upon co-application with glutamate simultaneously. Furthermore, the preincubation effects are less pronounced with partial agonists [22,23]. The differential effects due to pre-incubation provide indirect evidence for the state-dependent effect of Con-A. However, apart from these indirect measurements, the specific conformational mechanism underlying Con-A modulation is still unknown.

Here we used single-molecule fluorescence resonance energy transfer (smFRET) to study the conformational changes that underlie the reduction of desensitization in kainate receptors due to Con-A. FRET is ideally suited for such studies, as it can measure distances like a molecular ruler by detecting the extent of energy transfer between a donor and acceptor fluorophore, which are attached to specific sites on the protein of interest. Using the FRET methodology at the single-molecule level allows us to understand the conformational heterogeneity in the protein associated with each of the functional states [24,25,26,27,28,29,30,31,32,33,34]. We have previously used smFRET to investigate the conformations associated with the apo (unliganded), channel open/active, and desensitized states of the GluK2 homomeric kainate receptors [34]. These studies identified the ABD dimer interface as a site showing large-scale changes between active and desensitized states with no significant changes at the ATD interface across the subunits. The ABD dimer interface showed multiples states with varying degrees of decoupling, with the dimer interface being primarily tightly coupled in the resting and active states and shifting toward decoupled states upon desensitization [34]. This was consistent with the mechanism that was proposed based on cryo-EM structures of the resting and desensitized states [6,7,9]. Given that the subunit interfaces played a role in conformational control of function, here we studied the conformational changes underlying the reduction in desensitization of kainate receptors by Con-A by investigating the changes at the ATD and ABD subunit interfaces. Our studies show that Con-A alters the subunit interface at both the ATD and ABD favoring states that stabilize the active state of the receptor.

Prior structural and functional studies have shown that Na^+^ ions stabilize the dimer interface at the agonist-binding domain [10,35,36,37,38,39]. Removal of these ions, or their replacement with Cs^+^, leads to an increase in population of receptor conformations that are decoupled at the dimer interface, and this, in turn, favors inactivation and stabilization of the desensitized state of the receptor [34,37]. Given that Con-A binding favors the coupled dimer interface at the agonist-binding domain, we investigated the ion modulation function of kainate receptors in the presence of Con-A to determine if the two modulators had overlapping conformational control of function.

## 2. Methods

### 2.1. Generation of FRET Constructs

GluK2 constructs containing native glutamine at site 590 from *Rattus norvegicus* previously used in cryo-EM and smFRET studies were used. GluK2 insertion into pcDNA3.1; replacement of cysteine to serine at sites C91, C199, and C432; and mutation of site S266 (ATD) and A479 (LBD) to cysteine has been previously described in prior studies [6,7,33,34].

### 2.2. Electrophysiology

HEK 293T cells were transfected using Lipofectamine 2000 (GluK2-wt, GluK2-ABD, and GluK2-ATD) and co-transfected with GFP at a microgram ratio of 3:1. Whole-cell patch clamp recordings were performed 24–48 h after transfection using fire-polished borosilicate glass (Sutter instruments, Novato, CA, USA) pipettes with 3–5 megaohms resistance that were filled with internal solution: 110 mM CsF, 30 mM CsCl, 4 mM NaCl, 0.5 mM CaCl_2_, 10 mM HEPES, and 5 mM EGTA (adjusted to pH 7.4 with CsOH). The extracellular solution consisted of 150 mM NaCl or CsCl, 2.8 mM KCl, 1.8 mM CaCl_2_, 1.0 mM MgCl_2_, and 10 mM HEPES and was adjusted to pH 7.4 with NaOH or CsOH. External solutions were locally applied to lifted cells using a SF-77B perfusion fast-step (Warner Instruments, Holliston, MA, USA) in the presence or absence of 1 mM glutamate. Recordings were performed using an Axopatch 200B amplifier (Molecular Devices, San Jose, CA, USA) at −60 mV hold potential, acquired at 10 kHz using pCLAMP10 software (Molecular Devices), and filtered online at 5 kHz. Single-channel recordings were performed in the outside-out patch-clamp configuration 24 h after transfection (GluK2-wt). Patch pipettes had a resistance of 8 to 15 megaohms when filled with an internal solution. Buffers and solution concentrations were like those used for whole cell recordings. Data were acquired at 50 kHz and low pass filtered at 10 kHz (Axon 200B and Digidata 1550A; Molecular Devices). The holding potential was −100 mV. Data were further filtered at 1 Hz. All recordings were idealized using the segmental *k*-means algorithm of QuB [40,41].

### 2.3. smFRET Sample Preparation

HEK 293T cells were transiently transfected using the JetPrime protocol at 10 µg per 10-cm plate. One day after transfection, the cells from two 10-cm dishes were harvested; washed with extracellular buffer (ECB) containing 135 mM NaCl or CsCl, 3 mM KCl, 2 mM CaCl_2_, 20 mM glucose, and 20 mM HEPES; and adjusted to pH 7.4 with NaOH or CsOH. Post-wash, the cells were labeled for 1h at room temperature with 600 nM of donor fluorophore Alexa 555 maleimide (ThermoFisher, Waltham, MA, USA) and 2.4 µM of acceptor fluorophore Alexa 647 maleimide (ThermoFisher) in 3 mL of ECB. Post-labeling, the cells were washed and solubilized for 1 h at 4 °C. Solubilization buffer contained 1% lauryl maltose neopentyl glycol (Anatrace, Maumee, OH, USA), 2 mM cholesteryl hydrogen succinate (MP Biomedicals, Irvine, CA, USA), and ¼ protease inhibitor tablet (Pierce^™^) in phosphate-buffer saline containing either NaCl and Na_2_HPO_4_ for experiments in the presence of Na^+^ or CsCl and K_2_HPO_4_ for experiments in the absence of Cs^+^. Solubilized cells were filtered from unsolubilized debris by ultracentrifugation at 100,000× *g* for 1 h at 4 °C using a TLA 100.3 rotor. The supernatant was collected and kept on ice until used for smFRET samples.

### 2.4. smFRET Slide Preparation

Glass slides (20 × 20 mm) were washed using bath sonication in Liquinox phosphate-free detergent (Alconox, Inc., New York, NY, USA) followed by washing with 4.3% NH_4_OH and 4.3% H_2_O_2_ solution. Slides were then washed with purified water and dried with nitrogen gas. Post-drying, slides were plasma cleaned using a Harrick Plasma PDC-32G Plasma Cleaner followed by Vectabond (Vector Laboratories, Burlingame, CA, USA) treatment for aminosilanization. Slides were then stored under vacuum. Silicon templates (Grace Bio-Labs, Bend, OR, USA) were cleaned by sonication in methanol for 20 min followed by vortexing. Silicon templates were stored in clean methanol and dried with nitrogen gas when applied over Vectabond-treated slides. Slides were then treated with 50 µL of PEG solution containing 0.25% *w/w* biotinylated PEG, 25% *w/w* mPEG-succinimidyl carbonate, and either 0.1 M NaHCO_3_ or 0.1 m Tris for Na^+^ free conditions and stored overnight in a dark, moist environment. The following day, the slides were cleaned with purified water and dried with nitrogen gas prior to treatment with short-chain PEG solution containing 25 mM short-chain 333 Da MS(PEG)_4_ Methyl-PEG-NHS-Ester Reagent and either 0.1 M NaHCO_3_ or 0.1 M Tris, followed by room temperature incubation for 2–3 h. After incubation, slides were washed with purified water and dried with nitrogen gas. Silicon templates were removed, and Hybridwell chambers and press-fit tubing connectors (Grace Bio-Labs) were applied. Streptavidin solution containing 0.2 mg/mL streptavidin, 1X smFRET imaging buffer (1 mM DDM (n-dodecyl-β-d-maltoside), 0.2 mM CHS (cholesteryl hydrogen succinate), and 1X phosphate-buffer saline containing either NaCl and Na_2_HPO_4_ or CsCl and K_2_HPO_4_ was applied inside the chamber and incubated for 10 min, followed by washing with the appropriate 1X phosphate-buffer saline with or without Na^+^. 10 nM of biotinylated Goat Anti-Mouse IgG (H + L) secondary antibody (Jackson Immunoresearch Laboratories, Inc., West Grove, PA, USA, catalog number 115-065-003) was then flowed through the chamber and incubated for 20 min, followed by washing with the appropriate 1x phosphate-buffer saline. Next, 10 nM of 6X-His Tag mouse anti-Tag, Clone: HIS.H8 primary antibody (Invitrogen, Waltham, MA, USA, MA1213151MG) was introduced through the chamber and incubated for 20 min, followed by washing with the appropriate 1x phosphate-buffer saline. Bovine serum albumin (1 mg/mL) was introduced into the chamber and incubated for 15 min. Detergent-solubilized purified proteins containing 8X-His tagged homomeric GluK2 receptors were attached to the glass slide using an in situ immuno-precipitation (SiMPull) method by applying 50 µL of sample three times through the chamber and incubating for 20 min. 90 µL of oxygen-scavenging solution buffer system (ROXS) was applied inside the chamber containing 1 mM methyl viologen, 1 mM ascorbic acid, 0.01% *w/w* pyranose oxidase, 0.001% *w/v* catalase, 3.3% *w/w* glucose (all from Sigma-Aldrich, Inc., St. Louis, MO, USA), 1 mM DDM (Chem-Impex, Wood Dale, IL, USA), and 0.2 mM CHS (MP Biomedicals, LLC, Santa Ana, CA, USA) in phosphate-buffer saline containing either NaCl and Na_2_HPO_4_ or CsCl and K_2_HPO_4_, pH 7.4. In the glutamate-treated condition, 1 mM glutamate, 1 mM of MnCl_2_, and 1 mM of CaCl_2_ were introduced into the ROXS. In the Con-A treated condition, 40 µM Con-A (MilliporeSigma, Burlington, MA, USA), 1 mM of MnCl_2_, and 1 mM of CaCl_2_ were introduced into the ROXS and incubated for 10 min before application of glutamate-containing ROXS.

### 2.5. smFRET Data Acquisition

smFRET measurements were collected using a custom-built PicoQuant Microtime 200 Fluorescence Lifetime Microscope. To characterize the fluorescent behavior of both donor and acceptor fluorophores as well as the efficiency of the energy transfer between donors and acceptors, acquisition was conducted using both 532 nm (LDH-D-TA-530; Picoquant, Berlin, Germany) and 637 nm (LDH-D-C-640; Picoquant) lasers using pulsed interleaved excitation at 80 MHz. During scanning, the slide was immobilized on a scanning x-y-z piezo stage (P-733.2CD; Physik Instrumente) and observed through a 100× oil-immersed lens (100 × 1.4 NA; Olympus, Tokyo, Japan). Photons from samples post-excitation were then collected back through the objective and separated through a dual band dichroic beam splitter (Zt532/640rpc-UF3; AHF/Chroma, Bellows Falls, VT, USA). Prior to detection, photons were then filtered through emission filters (550 nm (FF01-582/64; AHF/Semrock, Rochester, NY, USA) for the donor or 650 nm (2XH690/70; AHF, Tübingen-Pfrondorf, Germany) for the acceptor) and into two SPAD photodiodes (SPCM CD3516H; Excelitas Technologies, Waltham, MA, USA).

### 2.6. smFRET Data Analysis

Molecules exhibiting a donor–acceptor anticorrelation and with a single photobleaching step were used in the analysis. FRET efficiencies were calculated based on the donor and acceptor intensities. MATLAB (Mathworks, Natick, MA, USA) was used to denoise donor and acceptor traces using wavelet-based denoising [42]. Origin (OriginLab Corp, Northampton, MA, USA) was used to create smFRET histograms and traces. The total numbers of molecules used for each condition were as follows: Con-A treated under Na^+^ condition, 40 molecules for GluK2-ATD and 38 molecules for GluK2-LBD; Con-A treated under Cs^+^ condition, 36 molecules for GluK2-LBD.

## 3. Results

**Figure 1:**
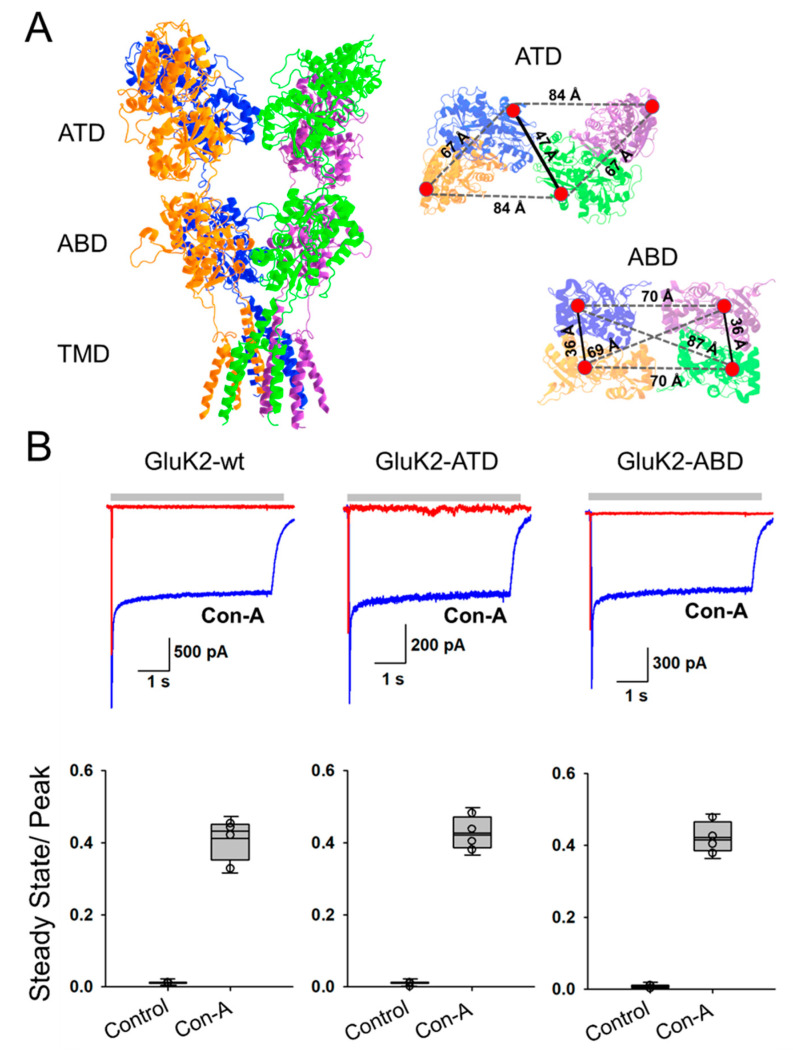
Depiction of the structural arrangement of homomeric GluK2 receptor and fluorophore conjugation sites. (**A**) Cryo-EM structure of a homomeric GluK2 receptor (PDB:5KUF) displaying the amino-terminal domain (ATD), agonist-binding domain (ABD), and transmembrane domain (TMD) and sites of fluorophore conjugation at the ATD (266) and ABD (479); top-down view shown for ATD (PDB:5KUF) and ABD (PDB:5KUH) with distances between sites 266 and 479, respectively. (**B**) Representative whole-cell recordings with 1 mM of glutamate for GluK2-wt, GluK2-ATD, and GluK2-ABD in the presence (blue) or absence (red) of 40 µM of Con-A (top) along with the ratio of steady-state to peak currents obtained from at least three different cells (bottom). These data show higher steady-state currents in the presence of Con-A.

We used maleimide-thiol reaction chemistry to attach donor and acceptor fluorophores at specific sites on the GluK2 receptor via mutated cysteines. To introduce cysteines at specific sites, we used a Cys-light GluK2 receptor, where the extracellular accessible cysteines at sites C91, C199, and C432 were mutated to serines [32,33,34]. For investigating changes at the dimer–dimer interface at the ATD we introduced a cysteine at site 266, henceforth referred to as GluK2-ATD. To study the interface within the dimer at the ABD, we introduced a cysteine at site 479 in the Cys-light background, henceforth referred to as GluK2-ABD. These sites were optimal for the donor (Alexa 555) and acceptor (Alexa 647) fluorophore pair, as FRET was only observed for the interface of interest, and all other distances had much lower FRET efficiencies (the R_0_ for Alexa 555 and Alexa 647 was 51 Å) (Figure 1). In order to attach the receptor to the imaging cover slip, we introduced an octa-histidine tag on the C-terminus of the receptor, allowing us to perform in situ pulldown of solubilized receptors that were expressed in HEK-293T cells [43]. Electrophysiological measurements were performed with the smFRET constructs, GluK2-ATD and GluK2-ABD, expressed in HEK-293T cells. The glutamate-mediated currents, as well as the reduction in the extent of desensitization by Con-A in the glutamate-mediated currents for these constructs, are shown in Figure 1 and were similar to that of the wild-type GluK2 receptor. This established that these smFRET constructs served as good models for wild-type GluK2 receptors.

**Figure 2:**
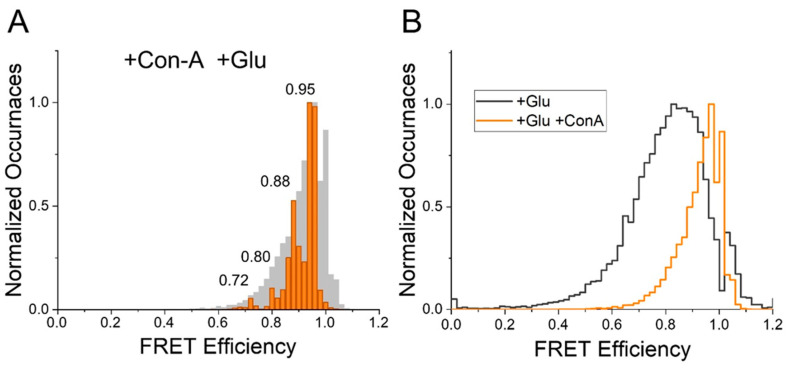
Conformational landscape of the distance across the dimer–dimer interface at the amino-terminal domain (ATD) of the homomeric GluK2 receptor (measured at site 266). (**A**) Denoised FRET efficiency histogram, in orange, overlapped onto an observed FRET efficiency histogram, in gray, depicting FRET efficiency occurrences in the presence of Con-A and glutamate. (**B**) Comparison between observed FRET efficiency histograms for glutamate-bound homomeric GluK2 receptors in the absence (in black) [34] and presence (in orange) of Con-A, illustrating a shift toward higher FRET efficiencies in the presence of Con-A.

**Figure 3:**
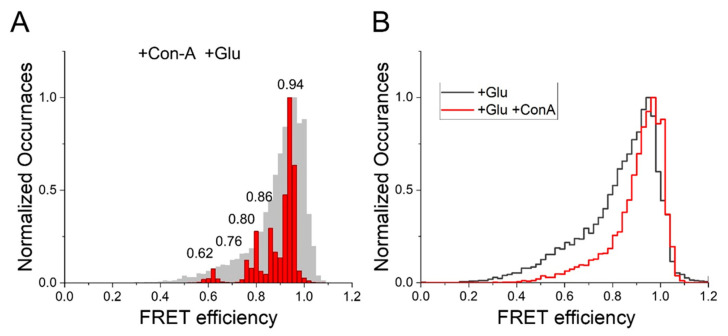
Conformational landscape of the distance between subunits at the dimer interface of the agonist-binding domain (ABD) of a homomeric GluK2 receptor (measured at site 479). (**A**) Denoised FRET efficiency histogram, in red, overlapped over observed FRET efficiency histogram, in gray, depicting FRET efficiency occurrences in the presence of Con-A and glutamate. (**B**) Comparison between observed FRET efficiency histograms of glutamate-bound homomeric GluK2 receptors in the absence (in black) [34] and presence (in red) of Con-A, illustrating a decrease in the low-FRET efficiency population in the presence of Con-A.

**Figure 4:**
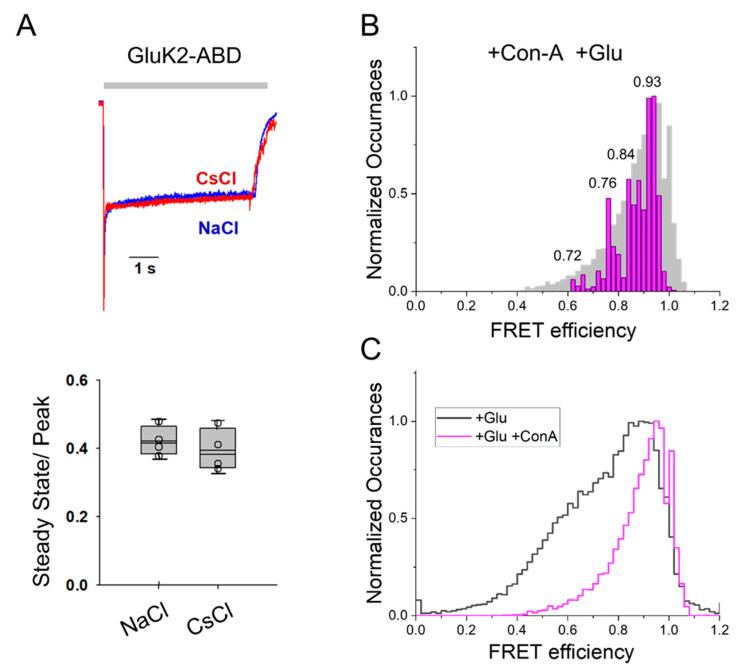
Effect of Con-A on ion modulation of GluK2 receptors. (**A**) Representative whole-cell recording with 1mM of glutamate and 40µM of Con-A for GluK2-ABD in the presence of either CsCl (red) or NaCl (blue) (top) along with the ratio of steady-state to peak currents obtained from at least three cells (bottom), showing no difference in steady-state response between NaCl- and CsCl-treated. (**B**) Denoised FRET efficiency histogram, in magenta, overlapped over the observed FRET efficiency histogram, in gray, depicting FRET efficiency occurrences in the presence of Con-A and glutamate obtained with CsCl replacing NaCl. (**C**) Comparison between observed FRET efficiency histograms of the homomeric, glutamate-bound, GluK2 receptor obtained with CsCl replacing NaCl, in the absence (in black) [34] and presence of Con-A (in magenta), illustrating a decrease in the low-FRET efficiency population in the presence of Con-A.

To perform smFRET measurements, we transfected HEK 293T cells with GluK2-ATD or GluK2-ABD and labeled them with donor and acceptor fluorophores. Detergent-solubilized membrane preparations of these cells were used for the in situ pull down of the receptor onto the coverslip. Fluorescence intensities of donor and acceptor fluorophores measured from single molecules using donor excitation (for determining FRET) were used to determine FRET efficiency between the donor and acceptor fluorophores. To ensure that only a single FRET distance was being measured, only traces exhibiting a single donor and a single acceptor photobleaching step and showing anticorrelation after the photobleaching of the acceptor were used to obtain FRET efficiencies. Representative single molecule traces and the ensemble FRET efficiency histograms from at least 30 molecules are shown in Figure 2 for GluK2-ATD and in Figure 3 and Figure 4 for GluK2-ABD. Wavelet-based denoising was performed on the donor and acceptor traces, and these denoised traces were used to determine FRET efficiencies of the different conformational states. Histograms showing the denoised efficiencies are overlaid on the histograms from the observed traces [42].

In the presence of Con-A and glutamate, the denoised smFRET efficiency histogram for GluK2-ATD showed that the receptor exhibited a primary population that had a FRET efficiency of 0.95, corresponding to the distance of 31 Å (Figure 2), with smaller fractions at FRET efficiencies of 0.88, 0.80, and 0.72, corresponding to distances of 37 Å, 40 Å, and 44 Å, respectively (Figure 2). The primary FRET efficiency of 0.95 for the ATD dimer distance, at site 266, was significantly higher than the primary FRET efficiency of 0.83 that we previously observed for GluK2-ATD receptors bound to glutamate in the absence of Con-A. This shift in the FRET efficiency population histogram is shown in the overlay of the observed FRET efficiency histogram for GluK2-ATD bound to glutamate in the presence and absence of Con-A in Figure 2. Based on these data showing conformations with higher FRET efficiency corresponding to shorter distances between the dimer–dimer subunits, we could conclude that Con-A binding to GluK2 receptors led to tighter packing at the ATD.

At the ABD dimer interface, smFRET histograms with GluK2-ABD receptors showed a major peak region at 0.94 FRET efficiency, corresponding to a distance of 32 Å (Figure 3) in the presence of Con-A and glutamate. Additional, less populous peaks were observed at 0.86, 0.80, 0.76, and 0.62, corresponding to 38 Å, 40 Å, 42 Å, and 47 Å, respectively (Figure 3). While conformations with similar FRET efficiencies were observed previously for the GluK2 receptors bound to glutamate in the absence of Con-A, the fraction of receptors in the high FRET states was significantly lower than that observed in the presence of Con-A. This shift in population to higher FRET efficiency states in the presence of Con-A can be seen in the overlay of the FRET efficiency histogram of GluK2-ABD bound to glutamate in the presence and absence of Con-A (Figure 3B). Prior structural and spectroscopic investigations of the α-amino-3-hydroxy-5-methyl-4-isoxazolepropionic acid receptor (AMPAR) and kainate receptors have shown that decoupling of the agonist-binding dimer drives desensitization in these receptors [6,9,44]. Thus, our data, which show a shift toward a more coupled dimer interface at the agonist-binding domain in the presence of Con-A, suggest that Con-A favors activation and reduces desensitization by allosterically stabilizing the dimer interface at the agonist-binding domain.

To further investigate the changes at the dimer interface of the ABD caused by Con-A binding, we studied the effect of Con-A on the Na^+^ modulation of kainate receptor function. For investigating Na^+^ ion modulation, we compared the whole-cell currents from GluK2-ABD homomeric receptors mediated by 10 mM glutamate in the presence of Con-A with Na^+^ ions in the extracellular buffer to those measured in the extracellular buffer when Na^+^ was replaced with Cs^+^ (Figure 4A). No significant differences were observed in the currents when Na^+^ was replaced with Cs^+^ in the presence of Con-A. These results in the presence of Con-A are in contrast with prior investigations showing a large decrease in currents when Na^+^ was replaced with Cs^+^ in the absence of Con-A [37]. Additionally, the extent of the residual current (non-desensitizing) due to stabilization with Con-A was similar in Cs^+^ (Figure 4A) and in Na^+^ (Figure 1). These investigations showed that in the presence of Con-A, the Na^+^ ion modulation of GluK2 receptor function was not observed and was consistent with the smFRET observations that showed that Con-A stabilized the ABD dimer interface and thus, removed the need for stabilization by Na^+^ ions. To further confirm that the stabilization of the dimer interface in the presence of Con-A was maintained in the presence of Cs^+^ ions, we investigated the distances at the dimer interface using smFRET. The smFRET histogram obtained with GluK2-ABD receptors when Na^+^ ions were replaced with Cs^+^ ions (Figure 4B) was similar to that obtained with Na^+^ ions (Figure 3). The shift to higher efficiency FRET conformations in the presence of Con-A relative to those previously reported in its absence in measurements performed when Na^+^ ions were replaced with Cs^+^ ions is shown by overlapping the FRET efficiency histograms (Figure 4C). Overall, the smFRET data show that Con-A favors a more coupled dimer interface even when Na^+^ ions are replaced with Cs^+^ ions, and this, in turn, can be related to the similar whole cell currents observed in both Na^+^ ions and Cs^+^ ions.

## 4. Discussion

Lectins such as Con-A are proteins that bind specifically to carbohydrates, glycoproteins, and glycolipids. The pharmacological effects of Con-A on kainate receptors have been studied extensively in both native and heterologous expression systems [16,17,18,19,36,45,46]. These studies have shown that Con-A increases the steady-state response of kainate receptors while not altering the kinetics for desensitization. To account for these electrophysiological observations, a kinetic mechanism involving multiple open states has been proposed, with Con-A shifting the population between these states. Additionally, mutational studies have shown that Con-A binds irreversibly to nine *N*-glycosylated amino acid residues located near the agonist-binding domain [18]. Given the multiple Con-A binding sites and the fact that tetrameric Con-A is more effective than the dimeric succinyl Con-A [38], it has been proposed that Con-A may act by restricting protein movements in the ABD. Consistent with the hypothesis that Con-A alters the agonist-binding domain, Con-A has been shown to have differential effects on currents mediated by agonists of differing efficacy [23]. These biochemical and functional studies provide indirect evidence of a shift in the conformational landscape of the agonist-binding domain as the mechanism underlying Con-A modulation of kainate receptors, and our smFRET investigations presented here provide insight into the specific conformations and shifts induced by Con-A binding.

Previous smFRET investigations allowed us to characterize the conformational landscape of the extracellular domain of GluK2 receptors in the apo (unliganded), glutamate-bound form (where the receptor was primarily desensitized) as well as the glutamate-bound D776K mutant receptor (where the receptor was primarily in the open active state) [34]. These studies showed that the dimer interface at the agonist-binding domain exhibits varying degrees of decoupling when the GluK2 receptor is bound to glutamate, while in the glutamate-bound form of the D776K mutant, the dimer interface of the agonist-binding domain exists primarily in a tightly coupled conformation. These studies suggested that the lower FRET (longer distance) decoupled states at the dimer interface of the ABD correlated to the desensitized state conformations, while the high FRET (shorter distance) coupled state at the dimer interface of the ABD represented the active, open-channel state of the receptor. The smFRET histograms for GluK2 receptors with Con-A at the dimer interface of the agonist-binding domain studied here showed a shift toward a higher FRET population, and the shift was consistent with the higher residual currents observed in the whole-cell recordings. These results suggest that modulation via Con-A is through a conformational selection process wherein conformation with the coupled dimer interface of the agonist-binding domain is favored.

This mechanism of Con-A modulation is further confirmed by the loss in Na^+^ ion modulation in the presence of Con-A. Na^+^ ions bind to the agonist-binding domain dimer interface and stabilize the interface. Replacing Na^+^ with Cs^+^ ions, which bind with a significantly lower affinity at the dimer interface, leads to lower currents and decreased stability of desensitized states in GluK2 receptors. This functional change is reflected in the higher population of lower FRET states in the presence of Cs^+^, further supporting the correlation between the decoupling at the dimer interface and the desensitization of the receptor. In this study, we showed that in the presence of Con-A, both function and conformational spread was unaltered when Na^+^ was replaced with Cs^+^ ions, as Na^+^ was no longer required for stabilization of the agonist-binding domain interface. Overall, our smFRET data showed a tighter packing at both the ATD and ABD subunit interfaces in the GluK2 receptor in the presence of Con-A that underlay the observed functional changes.

All ionotropic glutamate receptors are N-glycosylated, and deficiencies associated with glycosylation have been implicated in a number of diseases [47]. Interestingly, it has been shown that receptors without glycosylation still maintain their primary glutamate-gated ion channel function [17]. However, these studies were done using isolated receptors. The present study, along with prior functional studies, shows that lectins such as Con-A can modulate function by stabilizing extracellular domains upon binding to glycosylation sites. This raises the question as to whether endogenous proteins present in synapses can modulate ionotropic glutamate receptors by binding to glycosylation sites in a manner similar to what is seen for Con-A. This would imply that deficiencies in glycosylation might not affect isolated receptor function, but they would alter the modulatory effect of these proteins on receptor function in the context of the interacting proteins and thus, alter excitatory transmission. Recent studies have shown that several endogenous proteins, such as those that belong to the C1q/TNF superfamily, have a similar beta sheet fold as Con-A [48], and these have been shown to bind to ionotropic glutamate receptors. Among the most studied and better understood is cerebellin, an endogenous soluble protein, which forms a tripartite complex with ionotropic glutamate delta receptors and the presynaptic transmembrane protein, neurexin1β [49,50]. The modulatory mechanism through which these proteins work is still largely unknown. Future studies on the modulatory mechanism mediated by such C1q/TNF superfamily proteins, on the ionotropic glutamate receptors, and on the possible role of glycosylation on such modulation will shed light on whether these share common pathways to those observed here for Con-A binding to kainate receptors.

## Data Availability

The data generated, analyzed, and presented in this study are available from the corresponding author on reasonable request.
